# Glucose associated NETosis in patients with ST-elevation myocardial infarction: an observational study

**DOI:** 10.1186/s12872-019-1205-1

**Published:** 2019-10-15

**Authors:** Ragnhild Helseth, Eva Cecilie Knudsen, Jan Eritsland, Trine Baur Opstad, Harald Arnesen, Geir Øystein Andersen, Ingebjørg Seljeflot

**Affiliations:** 1 Center for Clinical Heart Research, Department of Cardiology, Oslo University Hospital Ullevål, PB 4956, Nydalen, 0424 Oslo, Norway; 2 University of Oslo, Oslo, Norway; 3 Department of Cardiology, Oslo University Hospital Ullevål, Oslo, Norway

**Keywords:** Acute ST-elevation myocardial infarction (STEMI), Neutrophil extracellular traps (NETs), Double-stranded deoxyribonucleid acid (dsDNA), Glucose, Glucometabolic status, Innate immunity, Neutrophil activation, Immunothrombosis

## Abstract

**Background:**

Neutrophil extracellular traps (NETs) have recently been identified as mediators in atherothrombosis. Although NETosis in general has been suggested to be glucose dependent, the transferability to patients with acute ST-elevation myocardial infarction (STEMI) is unclear. We assessed whether the NETs markers double-stranded deoxyribonucleid acid (dsDNA) and myeloperoxidase-DNA (MPO-DNA) associated with plasma glucose and the glucometabolic status in the acute phase and 3 months after a STEMI. We also explored whether an acute glucose load resulted in upregulated NETosis by assessment of peptidylarginine deiminase 4 (PAD4) gene expression.

**Methods:**

In total, 224 STEMI patients were prospectively enrolled and underwent blood sampling acutely (median 16.5 h after PCI) and after 3 months. Glucometabolic status was defined based on the results of an oral glucose tolerance test (OGTT) as normal glucose regulation (NGR), impaired fasting glucose (IFG), impaired glucose tolerance (IGT) or type 2 diabetes (T2DM). dsDNA and MPO-DNA were measured in serum, while PAD4 mRNA was measured in circulating leukocytes by RT-PCR.

**Results:**

dsDNA levels were significantly correlated to plasma glucose both acutely and after 3 months (*r* = 0.12 and *r* = 0.17, both *p* < 0.02), whereas MPO-DNA was not. No associations with the glucometabolic status were encountered for dsDNA and MPO-DNA acutely, but after 3 months dsDNA levels were elevated in patients with IFG and T2DM vs. NGR (428 vs. 371 ng/ml and 408 vs. 371 ng/ml, both *p* < 0.045). During the acute glucose load after 3 months, dsDNA and MPO-DNA remained unchanged while PAD4 mRNA increased significantly (RQ 0.836 vs. 0.920, *p* = 0.02).

**Conclusions:**

In this cohort of STEMI patients, levels of dsDNA associated with plasma glucose both in the acute and stable condition. The glucometabolic status was not substantially related to the selected NETs markers, however, an acute glucose load by OGTT performed after 3 months resulted in increased PAD4 expression, suggestive of enhanced NETosis in the aftermath of STEMI.

**Trial registration:**

www.clinicaltrials.gov, NCT00926133. Registered June 23, 2009.

## Background

Neutrophil extracellular traps (NETs), comprising fragments of neutrophil chromatin covered with neutrophil granule proteins extruding into the extracellular space upon neutrophil activation [1], have lately been identified as novel players in coronary artery disease (CAD). In stable CAD, levels of circulating NETs markers have been associated with CAD severity and prognosis [2, 3], while in acute ST elevation myocardial infarction (STEMI), levels of circulating NETs markers and the amount of NETs in aspirated coronary thrombi have both been associated with myocardial infarct size [4–6]. NETs probably enhance endothelial dysfunction, activate plasmacytoid dendritic cells and stimulate proinflammatory cytokine secretion from macrophages, which all drive atherosclerosis progression [7]. Substantial data also indicate that NETs are involved in thrombosis per se by serving as a scaffold for platelet entrapment and activation, coagulation activation and inbihition of fibrinolysis [7–11]. During NETs formation, NETosis, the enzyme peptidylarginine deiminase 4 (PAD4) catalyzes conversion of arginine residues of histones to citrulline in the neutrophil chromatin. This citrullination process results in histone charge change and chromatin decondensation, preceding neutrophil cell membrane rupture and NETs release, and is regarded a prerequisite for NETosis [12].

Multiple inducers of NETosis have been suggested, including gram positive and negative bacteria, fungi, lipopolysaccharide (LPS), proinflammatory interleukins, calcium channel opening agents and glucose [13]. NETosis has been reported to be glucose dependent in general and circulating NETs markers are shown to be elevated in patients with type 1 and 2 diabetes mellitus [14–19]. The metabolic requirements for NETosis in CAD have, however, not been addressed. In the present investigation we aimed to explore whether the circulating NETs markers double stranded deoksyribonucleic acid (dsNDA) and myeloperoxidase-DNA (MPO-DNA) are associated with plasma glucose and the glucometabolic status in a cohort of STEMI patients. We further explored whether an acute glucose load by oral glucose tolerance test (OGTT) 3 months after the STEMI had any impact on NETosis, assessed by PAD4 messenger ribonucleic acid (mRNA) levels in circulating leukocytes as well as serum levels of dsDNA and MPO-DNA. Our hypothesis was that hyperglycemia would act as a NETosis stimulating agent in STEMI patients, thus contributing to increased understanding of NETs in CAD. If NETs have a negative impact on myocardial infarct size, identification of NETosis stimulating agents may participate in paving the way for directed anti-NETs therapy.

## Methods

### Study population

This is a substudy of the” Abnormal Glucose Regulation in Patients with Acute Myocardial Infarction” trial, and details of the study cohort have previously been published [20]. In brief, 224 prospectively enrolled patients with STEMI treated with primary percutaneous coronary intervention (PCI) at Ullevål University Hospital, Oslo, Norway during 2005 – 2007 were investigated for the prevalence of abnormal glucose regulation (AGR) in the acute phase and the reliability of an OGTT performed early after the acute event to predict AGR 3 months later. Patients with clinical instability, known diabetes or persistent hyperglycemia (defined as combination of admission glucose > 11 mmol/L and fasting glucose level > 8 mmol/L), serum creatinine concentration ≥ 200 μmol/L or age > 85 years were excluded.

### Laboratory analyses

Blood samples were drawn after an overnight fasting in the acute phase at a median of 16.5 h (h) after hospital admission and repeated after 3 months. Standardized OGTT (2 h) with 75 g glucose dissolved in 200 ml water [21] was performed at both time points (*n* = 224 in the acute phase, *n* = 201 after 3 months) in order to determine the glucometabolic status as defined by the World Health Organization (WHO) [22]. dsDNA levels (ng/ml) were quantified in serum by Quant-iT Picogreen dsDNA Assay # P11496 (Invitrogen, Carlsbad, CA, USA). MPO-DNA complexes were quantified in serum by an enzyme-linked immunosorbent assay (ELISA) technique originally described by Kessenbrock et al. [23]. Briefly, plates were coated with anti-MPO (AbD Serotec, Hercules, CA, USA) and incubated overnight at 4 °C. After blocking with bovine serum albumine (BSA), patient serum and a peroxidase-labeled anti-DNA antibody (Cell Death Detection kit, Roche Diagnostics GmbH, Mannheim, Germany) were added. After 2 hours of incubation, a peroxidase substrate was added and absorbance was measured after 40 min. Data are reported as optical density (OD) units. The inter assay CVs for dsNDA and MPO-DNA were 8.2 and 8.4%, respectively.

PAXGene Blood RNA tubes were collected for RNA extraction from circulating leukocytes in a randomly selected subset of 100 patients at 3 months. Total RNA was reversely transcribed into complementary DNA (cDNA) by use of qScript cDNA SuperMIx (Quanta Biosciences, Inc.,Gaithersburg, USA). Expression of the PAD4 gene was assessed by real time polymerase chain reaction (RT PCR) on the VIIa7 Instrument (Applied Biosystems, by Life Technologies, Foster City CA, USA), using TaqMan Universal PCR Master Mix, No AmpErase UNG and the PAD4 TaqMan assay (Hs01057483_m1), and measured as relative quantification (RQ) (2 ^–ΔΔCt^ method) [24], with beta-2-microglobulin (β_2_M) as house-keeping gene (Assay ID Hs99999907_m1) (all Applied Biosystems).

### Definition of glucometabolic status

Glucometabolic status based on OGTT was defined according to the World Health Organization criteria [22] (Table 1). Due to limited number in each category, patients were also classified into normal vs. abnormal glucose regulation (NGR/AGR), the latter being the sum of impaired fasting glucose (IFG), impaired glucose tolerance (IGT) and type 2 diabetes (T2DM).

**Table 1:** Glucometabolic status

	Glucose level (0 min)		Glucose level (2 h)	Acute phase	3 months
NGR	< 6.1	and	< 7.8	119/224 (53.1%)	151/201 (75.1%)
IFG	≥ 6.1 < 7.0	and	< 7.8	12/224 (5.4%)	12/200 (6.0%)
IGT	< 7.0	and	≥ 7.8 < 11.1	69/224 (30.8%)	29/200 (14.5%)
T2DM	≥ 7.0	and/or	≥ 11.1	24/224 (10.7%)	10/201 (5.0%)
AGR	IFG + IGT + T2DM			105/224 (46.9%)	50/201 (24.9%)

Glucose levels are given in mmol/L. Numbers (%) are given for the separate glucometabolic categories in the acute phase and after 3 months. *AGR* abnormal glucose regulation, *NGR* normal glucose regulation, *IFG* impaired fasting glucose, *IGT* impaired glucose tolerance, *T2DM* type 2 diabetes mellitus

### Statistics

As the majority of relevant variables were skewed distributed, non-parametric statistics were used throughout. Descriptive data are given by medians (25, 75 percentiles) or numbers (%), as appropriate. Correlation analyses were performed by Spearman’s rho. Differences between groups were assessed by the Mann-Whitney U test, while time-dependent changes were analyzed by Friedman’s test followed by Wilcoxon Signed Rank test. A *p* value ≤0.05 was considered statistically significant. All statistical analyses were performed in IBM SPSS Statistics, version 25.

## Results

### General cohort characteristics

**Fig. 1:**
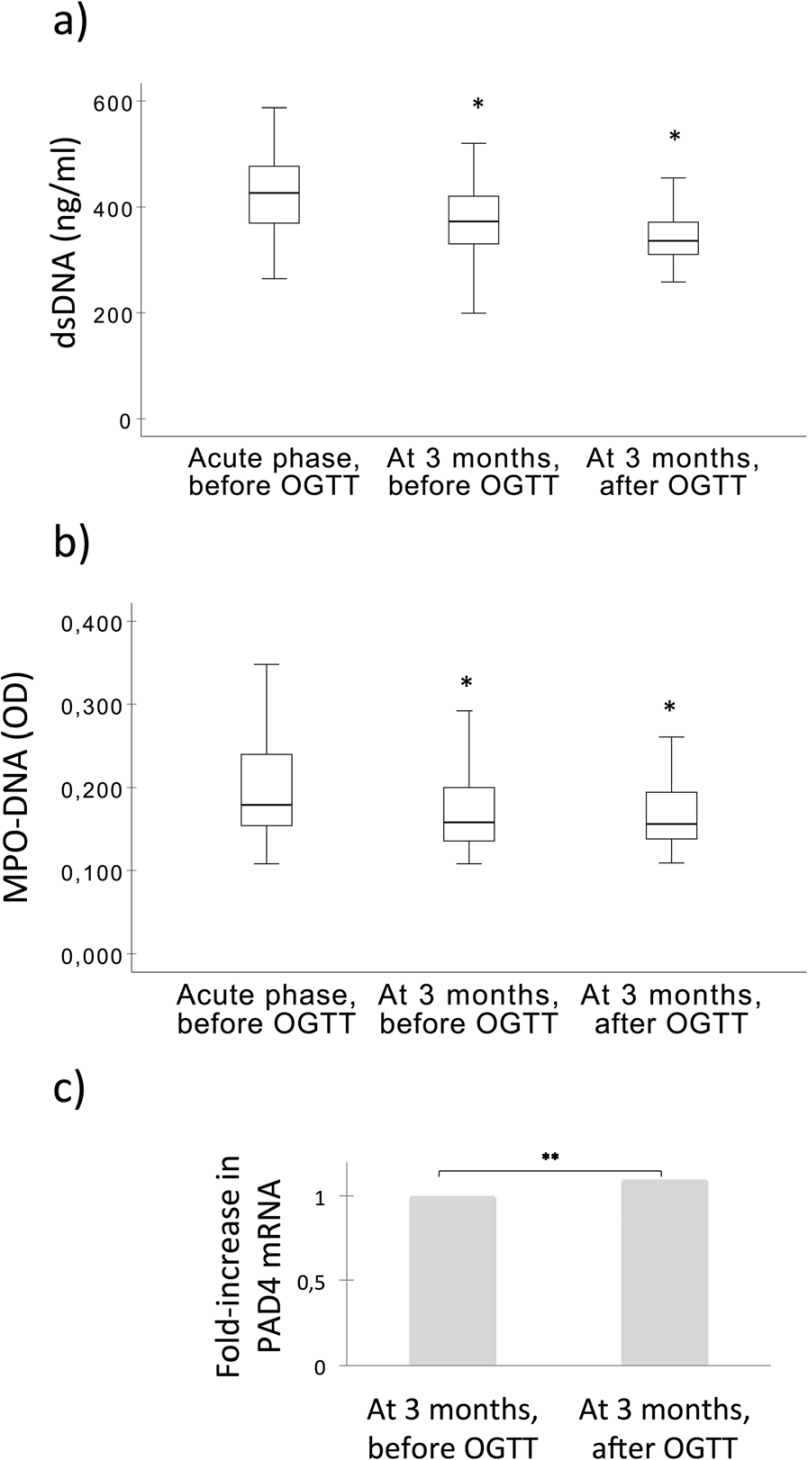
The time profiles of NETs markers and change during OGTT. **a** dsDNA, **b** MPO-DNA, **c** PAD4 mRNA. Values are given as medians (25, 75 percentiles). *p* values are based on the Wilcoxon Signed Rank test. *: Significant change from the acute phase. **: Significant change during the course of OGTT. dsDNA: double-stranded deoxyribonucleic acid. MPO-DNA: myeloperodidase deoxyribonucleid acid. OGTT: oral glucose tolerance test. OD: optical density units. PAD4 mRNA: peptidyl arginine deiminase 4 messenger ribonucleic acid

The included patients had a median age of 58 years and the majority was males (Table 2). Approximately 50% were current smokers, 25.9% had hypertension, 8.9% had hyperlipidemia and 7.1% had previously undergone a myocardial infarction (MI). Median leukocyte count at the time of hospital admission was 10.2 ×  10^9^/L. Peak troponin T was 4.7 μg/L. As previously reported and outlined in Tables 1 and 2, the prevalence of AGR in the acute phase was 46.9% [20]. At 3 months, 201 of the patients repeated the OGTT with a prevalence of AGR of 24.9% [20]. Levels of dsDNA and MPO-DNA were both significantly higher in the acute phase than after 3 months (both *p* < 0.01) (Fig. 1a and b).

**Table 2:** Characteristics of the study population

Age (years)	58	(51,67)
Female gender	39	(17.4)
BMI (kg/m^2^)	26	(24.4,28.7)
Waist circumference (cm)	100	(94,107)
Current smoking	109	(48.7)
Previous medical history		
Hypertension	58	(25.9)
Hyperlipidemia	20	(8.9)
Myocardial infarction	16	(7.1)
Acute phase		
Leukocyte count (× 10^9^/l)	10.2	(8.5,12.4)
Ischemic time (min)^a^	219	(139,382)
HbA1c (%)	5.5	(5.3,5.8)
Plasma glucose, start of OGTT (mmol/L)	5.3	(4.9,5.9)
Plasma glucose, end of OGTT (mmol/L)	7.3	(5.9,8.8)
AGR	105	(46.9)
Peak Troponin T (μg/L)	4.7	(2.4,9.0)
Medication at hospital discharge		
Acetylsalicylic acid	224	(100)
Clopidogrel	222	(99.1)
Statins	221	(98.7)
Betablockers	181	(80.8)
ACE-inhibitors	36	(16.1)
ATII antagonists	18	(8.0)
After 3 months		
Plasma glucose, start of OGTT (mmol/L)	5.2	(4.8,5.6)
Plasma glucose, end of OGTT (mmol/L)	5.4	(4.5,7.1)
AGR	50	(24.9)
Infarct size, %^b^	14	(0,29)
LVEF, %^b^	64	(56,70)

Values are given as medians (25, 75 percentiles) or numbers (%) as appropriate. ^a^Defined as time form symptom debut to PCI. ^b^Assessed by Single photon emission computed tomography (SPECT). *ACE* angiotensin converting enzyme, *AGR* abnormal glucose regulation, *ATII* angiotensin II, *LVEF* left ventricular ejection fraction, *OGTT* oral glucose tolerance test

### Correlations between NETs markers and leukocytes

dsDNA and MPO-DNA levels were significantly correlated to leukocyte count at admission (*r* = 0.186 and *r* = 0.245, *p* < 0.01 for both) and to each other in the acute (*r* = 0.269, *p* < 0.001), but not the stable phase after 3 months.

### Associations to plasma glucose and glucometabolic status

**Fig. 2:**
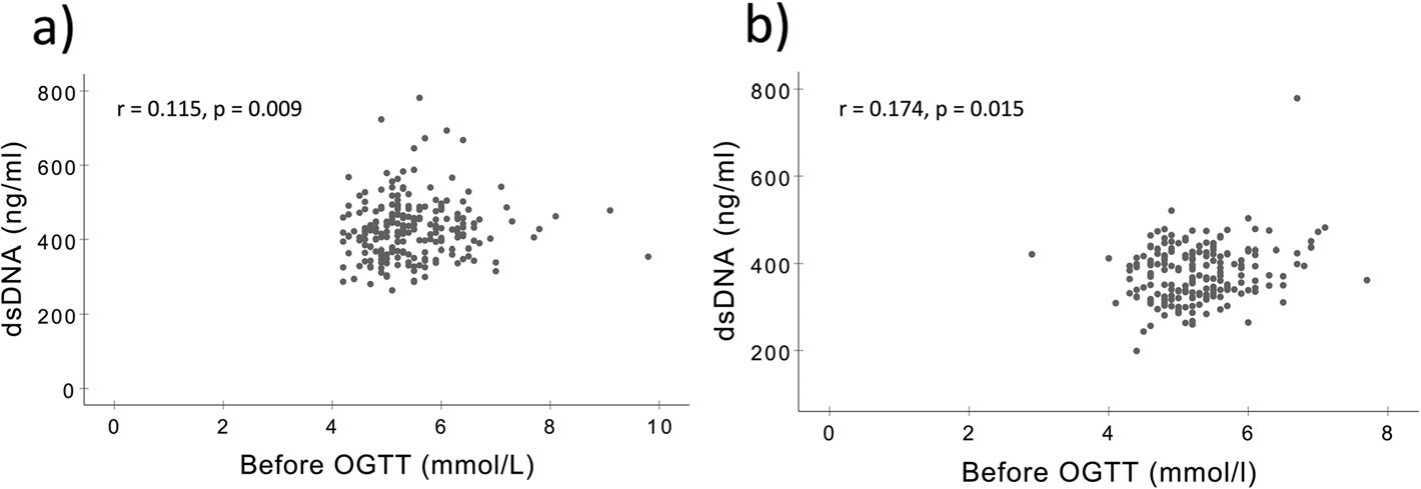
Correlation between glucose and dsDNA. **a** Acute phase, **b** at 3 months. *p* values are based on the Spearman’s rho. dsDNA: double-stranded deoxyribonucleic acid. OGTT: oral glucose tolerance test

**Fig. 3:**
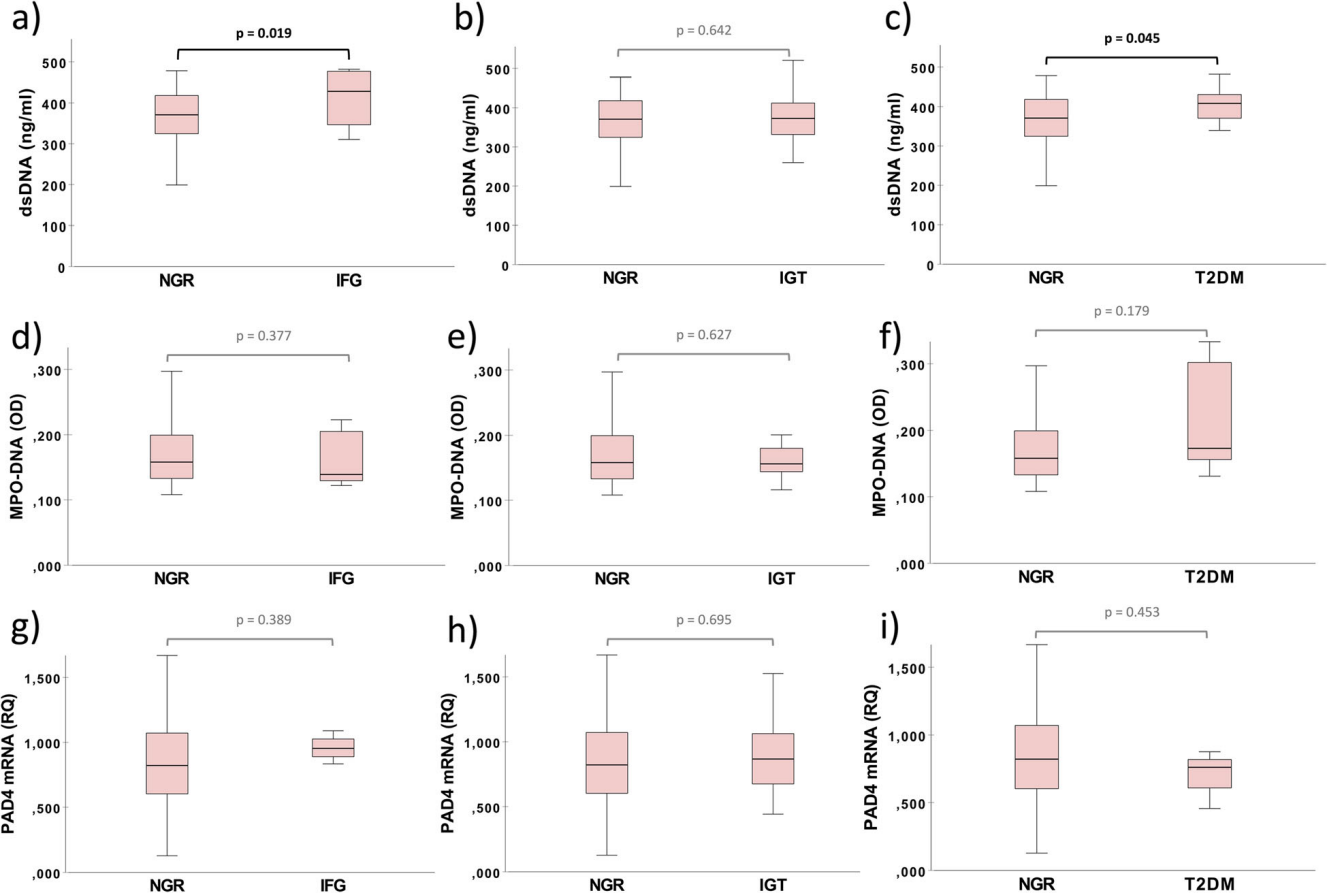
NETs markers related to the glucometabolic status at 3 months. Values are given as medians (25, 75 percentiles). **a**-**c**: dsDNA levels in NGR vs. IFG, IGT and T2DM, respectively. **d**-**f**: MPO-DNA levels in NGR vs. IFG, IGT and T2DM, respectively. **g**-**i**: PAD4 mRNA levels in NGR vs. IFG, IGT and T2DM, respectively.*p* values are based on the Mann-Whitney U test. dsDNA: double-stranded deoxyribonucleic acid. IFG: impaired fasting glucose. IGT: impaired glucose tolerance. MPO-DNA: myeloperoxidase deoxyribonucleic acid. NGR: normal glucose regulation. OD: optical density units. PAD4 mRNA: peptidylarginine deiminase 4 messenger ribonucleic acid. RQ: relative quantification values. T2DM: type 2 diabetes mellitus

dsDNA levels correlated weakly, but significantly to plasma glucose before OGTT both in the acute phase and at 3 months (Fig. 2). No significant correlations were encountered between plasma glucose and MPO-DNA and PAD4 mRNA levels (Additional file 1: Table S1). In the acute phase, levels of dsDNA and MPO-DNA did not differ significantly between patients with NGR vs. AGR, or between patients with NGR vs. IFG, NGR vs. IGT, or NGR vs. T2DM when these components of AGR were investigated separately (Table 3, Additional file 2: Table S2). After 3 months, dsDNA was borderline significantly higher in patients with AGR vs. NGR, whereas MPO-DNA and PAD4 mRNA levels did not differ (Table 3). When comparing the AGR components separately after 3 months, dsDNA levels were significantly higher in patients with IFG vs. NGR (*p* = 0.019) and in patients with T2DM vs. NGR (*p* = 0.045), whereas levels of MPO-DNA and PAD4 mRNA did not differ between the three AGR categories (Fig. 3).

**Table 3:** NETs markers related to NGR vs. AGR

	NGR	AGR	p
Acute phase			
dsDNA (ng/ml)	425 (370,467)	429 (367,481)	0.491
MPO-DNA (OD)	0.179 (0.153,0.248)	0.180 (0.155,0.236)	0.752
After 3 months			
dsDNA (ng/ml)	371 (325,418)	394 (343,434)	0.052
MPO-DNA (OD)	0.158 (0.133,0.200)	0.156 (0.140,0.209)	0.545
PAD4 mRNA (RQ)	0.822 (0.603,1.075)	0.872 (0.704,1.009)	0.693

*p* values are based on the Mann-Whitney U test. Values are given as medians (25, 75 percentiles). *AGR* abnormal glucose regulation, *dsDNA* double-stranded deoxyribonucleid acid, *MPO-DNA* myeloperoxidase deoxyribonucleid acid, *NGR* normal glucose regulation, *OD* optical density units, *PAD4 mRNA* peptidylarginine deiminase 4 messenger ribonucleid acid

### NETs markers during OGTT

While neither dsDNA nor MPO-DNA levels changed significantly during the course of OGTT at 3 months (Fig. 1a and b), PAD4 mRNA levels in circulating leukocytes increased 1.1 fold during the acute glucose load (RQ 0.836 vs. 0.920, *p* = 0.02) (Fig. 1c).

## Discussion

While the glucometabolic status overall only modestly influenced serum levels of NETs markers in this cohort of STEMI patients, plasma glucose per se was associated with levels of dsDNA both in the acute phase and in the stable condition after 3 months. An acute glucose load by OGTT performed 3 months after the STEMI resulted in increased gene expression of PAD4, suggestive of enhanced NETosis, although not accompanied by an increase in dsDNA or MPO-DNA levels. The latter might be caused by a delayed time course of NETs release.

The observed positive correlation between serum levels of dsDNA and plasma glucose is in line with existing literature suggesting that NETosis might be glucose dependent [14, 15, 17, 18]. Nevertheless, such association has not previously been specifically addressed in patients with acute MI, in which acute hyperglycemia has been associated with poorer outcome [25, 26]. It is interesting to note that the mechanisms linking acute hyperglycemia to poorer outcome in MI, eg. generation of reactive oxygen species (ROS), enhanced platelet and coagulation cascade activation, endothelial dysfunction and upregulated inflammation [26] overlap with suggested NETs mediated effects. Thus, the association between circulating dsDNA and plasma glucose in this cohort of patients with STEMI could shed light on how hyperglycemia is linked to adverse effects in acute MI. The absence of any association between the NETs markers and the glucometabolic status in the acute setting in our study is not easily discussed along with existing literature. However, higher levels of dsDNA were observed in patients with IFG and T2DM compared to patients with NGR after 3 months, which reflect a more stady state. The somewhat inconsistency could be due to the fact that patients with known diabetes and therefore more longlasting hyperglycemia were excluded from the study. Thus, the patients diagnosed with glucometabolic disturbances were probably in an “early” stage of their tendency towards hyperglycemia. It has, nevertheless, also been reported that neutrophils may become insensitive to NETs inducing stimuli during chronic hyperglycemia [27], which probably were the case for many of the patients with glucometabolic disturbances in this cohort. Based on our observations, however, NETs might be associated with the glucometabolic status in the more stable CAD setting. The complex pathophysiology during the acute course of a MI does not make it unlikely that the regulation of NETs and NETosis differ along the time axis from the acute event. Underpinning the latter, dsDNA and MPO-DNA levels were both significantly higher in the acute phase than after 3 months.

**Fig. 4:**
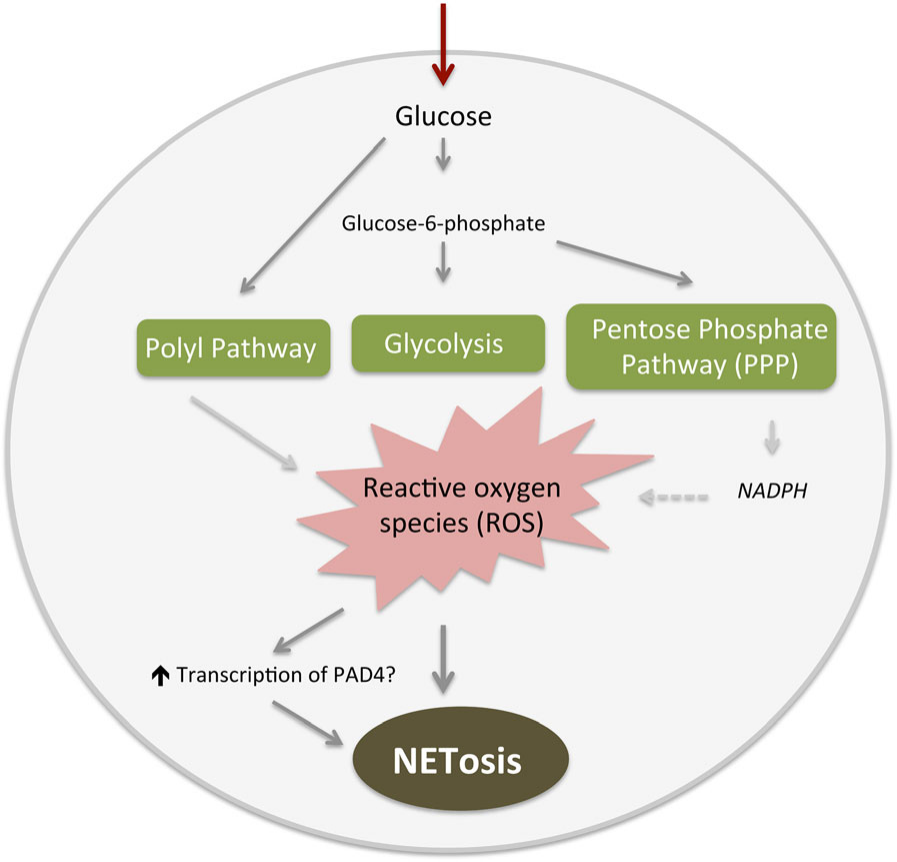
Suggested metabolic pathways involved in glucose mediated NETosis, simplified schematic illustration

Gene expression levels of the NETosis related enzyme PAD4 increased significantly during the acute glucose load by OGTT after 3 months. The increase in PAD4 was not accompanied by increased levels of dsDNA or MPO-DNA. As the time course of NETs release upon different inducers of NETosis in CAD is unknown, this observation may imply that an acute glucose load in a stable state after STEMI is associated with NETosis. Although not reported in CAD, different metabolic intracellular signaling pathways have been suggested to be involved in the link between glucose and NETosis (Fig. 4). First, inhibition of glycolysis has been reported to inhibit NETosis [15]. Second, NETs formation depends on generation of reactive oxygen species (ROS) and intracellular hyperglycemia increases ROS production in neutrophils [28–30]. Third, the pentose phosphate pathway generates NADPH that might be used as a cofactor in ROS production [31]. Fourth, inhibition of the polyl pathway has been reported to abbrogate NETs formation completely [17]. Exessive ROS per se have also been reported to increase transcription and activation of PAD4 directly by increasing intracellular Ca^++^ concentration [29]. It has moreover been reported that acute glucose fluctuations, like during OGTT, rather than chronic hyperglycemia trigger ROS production [32]. The latter could be discussed in line with our observation of modest association between the NETs markers and the glucometabolic status, representing a more chronic situation. Taken together, the observed upregulation of PAD4 as a marker of NETosis during an acute oral glucose load is well in line with current literature and indicate that NETosis in the stable setting after a STEMI might be upregulated by acute fluctuations in plasma glucose levels. Further work to verify our observations and to explore details of the intracellular pathways linking hyperglycemia to NETosis in patients with CAD and acute MI is warranted. It also remains to be determined whether the observed associations between plasma glucose and markers of NETosis potentially can be translated into meaningful clinical applications.

### Limitations

The observational nature of the study impeedes clear-cut conclusions. The results are nevertheless hypothesis generating. The discrepancy between the observations for dsDNA and MPO-DNA is worth noticing as these markers currently both are regarded as relatively solid surrogate markers of NETs [33]. We recently reported on levels of dsDNA, but not MPO-DNA, to be associated with clinical outcome in patients with stable CAD, underpinning the mismatch between these assumed NETs markers [3]. Whether this issue reflects that dsDNA and MPO-DNA do not origin from the same cellular source, that NETs in patients with CAD cannot be compared to NETs in other disease entities as to the content, concentration and distribution of neutrophil proteins, or whether other physiological or methodological aspects could be relevant are unclear and in need of further exploration.

The spesificity of dsDNA as a marker of NETs can also be discussed, particularily in STEMI patients where myocardial cell death probably contributes to the amount of extracellular nuclear material. The PAD4 gene expression analyses were performed in leukocytes, and not specifically in neutrophils, which might have influenced the results. Finally, the fact that not all forms of NETosis are PAD4 dependent complicates interpretation of the current observations [34].

## Conclusions

In this cohort of STEMI patients, plasma glucose correlated to the NETs marker dsDNA both in the acute phase and after 3 months. Although the glucometabolic status overall was only modestly associated with the selected NETs markers, an acute oral glucose load after 3 months resulted in upregulated PAD4 gene expression, suggestive of enhanced NETosis. Potential modulators of NETosis, like plasma glucose, might become clinically relevant in the quest to influence adverse effects of NETosis in CAD.

## Supplementary information

**Additional file 1: Table S1.** Correlations between NETs markers and plasma glucose levels. *p* values are based on the Spearman’s rho. dsDNA: double-stranded deoxyribonucleic acid. MPO-DNA: myeloperoxidase deoxyribonucleic acid. PAD4 mRNA: peptidylarginine deiminase 4 messenger ribonucleic acid. OD: optical density units. r: correlation coefficient. RQ: relative quantification values.

**Additional file 2: Table S2.** dsDNA and MPO-DNA related to the glucometabolic status in the acute phase. Values are given as median (25, 75 pencentiles). *p* values are based on the Mann-Whitney U test. dsDNA: double-stranded deoxyribonucleic acid. IFG: impaired fasting glucose. IGT: impaired glucose tolerance. MPO-DNA: myeloperoxidase deoxyribonucleic acid. OD: optical density units. T2DM: type 2 diabetes mellitus. p^a^: NGR vs. IFG. p^b^: NGR vs. IGT. p^c^: NGR vs. T2DM.

## Data Availability

The datasets used and/or analysed during the current study are available from the corresponding author on reasonable request.

## Abbreviations

ACE: Angiotensin converting enzyme
AGR: Abnormal glucose regulation
ATII: Angiotensin II
BSA: Bovine serum albumine
CAD: Coronary artery disease
dsDNA: Double-stranded deoxyribonucleic acid
IFG: Impaired fasting glucose
IGT: Impaired glucose tolerance
LPS: Lipopolysaccharide
LVEF: Left ventricular ejection fraction
MPO-DNA: Myeloperoxidase-deoxyribonucleic acid
mRNA: Messenger ribonucleic acid
NADPH: Reduced form of nicotinamide adenine dinucleotide phosphate
NETs: Neutrophil extracellular traps
NGR: Normal glucose regulation
OD: Optical density units
OGTT: Oral glucose tolerance test
PAD4: Peptidylarginine deiminase 4
PCI: Percutaneous coronary intervention
REK: Regional ethics committee
ROS: Reactive oxygen species
RQ: Relative quantification values
RT PCR: Real time polymerase chain reaction
SPECT: Single photon emission computed tomography
STEMI: ST-elevation myocardial infarction
T2DM: Type 2 diabetes mellitus
β_2_M: Beta-2-microglobulin
