# Supplementary material for: Glucose associated NETosis in patients with ST-elevation myocardial infarction: an observational study
Source: BMC Cardiovasc Disord. 2019 Oct 15;19:221. doi: 10.1186/s12872-019-1205-1 (PMC6794742; doi:10.1186/s12872-019-1205-1)
Supplement: Supplementary file 1 — Additional file 1: Table S1. Correlations between NETs markers and plasma glucose levels. p values are based on the Spearman’s rho. dsDNA: double-stranded deoxyribonucleic acid. MPO-DNA: myeloperoxidase deoxyribonucleic acid. PAD4 mRNA: peptidylarginine deiminase 4 messenger ribonucleic acid. OD: optical density units. r: correlation coefficient. RQ: relative quantification values. [file 12872_2019_1205_MOESM1_ESM.doc]

**Table S1** Correlations between NETs markers and plasma glucose levels

|  |  |  | r | p |
| --- | --- | --- | --- | --- |
| dsDNA (ng/ml) | Acute phase |  | **0.115** | **0.009** |
|  | After 3 months | Before OGTT | **0.174** | **0.015** |
|  |  | After OGTT | -0.08 | 0.407 |
| MPO-DNA (OD) | Acute phase |  | 0.020 | 0.769 |
|  | After 3 months | Before OGTT | -0.078 | 0.285 |
|  |  | After OGTT | 0.147 | 0.123 |
| PAD4 mRNA (RQ) | Acute phase |  | - | - |
|  | After 3 months | Before OGTT | 0.056 | 0.577 |
|  |  | After OGTT | 0.048 | 0.636 |

p values are based on the Spearman´s rho test. dsDNA: double-stranded deoxyribonucleic acid. MPO-DNA: myeloperoxidase deoxyribonucleic acid. PAD4 mRNA: peptidylarginine deiminase 4 messenger ribonucleic acid. OD: optical density units. r: correlation coefficient. RQ: relative quantification values.
