# Supplementary material for: Glucose associated NETosis in patients with ST-elevation myocardial infarction: an observational study
Source: BMC Cardiovasc Disord. 2019 Oct 15;19:221. doi: 10.1186/s12872-019-1205-1 (PMC6794742; doi:10.1186/s12872-019-1205-1)
Supplement: Supplementary file 2 — Additional file 2: Table S2. dsDNA and MPO-DNA related to the glucometabolic status in the acute phase. Values are given as median (25, 75 pencentiles). p values are based on the Mann-Whitney U test. dsDNA: double-stranded deoxyribonucleic acid. IFG: impaired fasting glucose. IGT: impaired glucose tolerance. MPO-DNA: myeloperoxidase deoxyribonucleic acid. OD: optical density units. T2DM: type 2 diabetes mellitus. pa: NGR vs. IFG. pb: NGR vs. IGT. pc: NGR vs. T2DM. [file 12872_2019_1205_MOESM2_ESM.doc]

**Table S2** dsDNA and MPO-DNA related to the glucometabolic status in the acute phase

|  | NGR | IFG | IGT | T2DM | p^a^ | p^b^ | p^c^ |
| --- | --- | --- | --- | --- | --- | --- | --- |
|  |  |  |  |  |  |  |  |
| dsDNA (ng/ml) | 425 (370, 467) | 410 (365, 452) | 434 (364, 484) | 427 (380, 490) | 0.416 | 0.457 | 0.364 |
| MPO-DNA (OD) | 0.179 (0.153, 0.248) | 0.161 (0.143, 0.222) | 0.191 (0.154, 0.238) | 0.178 (0.157, 0.201) | 0.263 | 0.880 | 0.732 |

p values are based in Mann-Whitney U test. Values are given as median (25, 75 pencentiles). dsDNA: double-stranded deoxyribonucleic acid. IFG: impaired fasting glucose. IGT: impaired glucose tolerance. MPO-DNA: myeloperoxidase deoxyribonucleic acid. OD: optical density units. T2DM: type 2 diabetes mellitus. p^a^: NGR vs. IFG. p^b^: NGR vs. IGT. p^c^: NGR vs. T2DM.
